# A rational emotive behavior therapy-based intervention for binge eating behavior management among female students: a quasi-experimental study

**DOI:** 10.1186/s40337-020-00347-8

**Published:** 2020-12-02

**Authors:** Jiwon Yang, Kuem Sun Han

**Affiliations:** 1 Department of Nursing, Kyungil University, Gyeongsan-si, South Korea; 2 Department of Nursing, Korea University, Seoul, South Korea

**Keywords:** Female, Students, Binge eating disorder, Self-criticism, Narcissism, Perfectionism, Body dissatisfaction, Anxiety, Depression

## Abstract

**Background:**

Binge eating behavior is highly likely to progress to an eating disorder, with female students particularly at risk.

**Objective:**

This study aimed to verify the effect of a binge eating behavior management program, based on rational emotive behavior therapy (REBT), on binge eating behavior and related cognitive and emotional factors among female college students.

**Method:**

The study, conducted from November 1 to December 2, 2016, involved a pretest-posttest design and nonequivalent control group. The sample included 24 and 22 first- to third-year students, from a college in South Korea, in the experimental and control groups, respectively. Data were collected using self-esteem, covert narcissism, perfectionism, body dissatisfaction, anxiety, depression, and binge eating scales and analyzed via frequency analysis, χ^2^ tests, t tests, and analysis of covariance.

**Results:**

The results indicated that the REBT-based binge eating behavior management program exerted positive effects on participants’ self-esteem, reducing covert narcissism, body dissatisfaction, anxiety, depression, and binge eating. However, there was no significant difference in perfectionism, although the experimental group’s mean score decreased from pretest to posttest.

**Conclusions:**

Based on the results, the program was considered to be effective, and is expected to be useful in preventing the development of eating disorders among female college students by treating binge eating behavior and related cognitive and emotional factors. This intervention could ultimately contribute to the improvement of female college students’ health and quality of life.

## Plain English summary

Binge eating behavior results from an individual’s distorted body image, dissatisfaction with their physical appearance, and idealization of thin body types, and requires intervention before developing into an eating disorder. The purpose of this study was to address binge eating behavior and related factors among female college students in Korea, ultimately aiming to prevent the development of eating disorders. Our intervention integrated cognitive, emotional, and behavioral techniques used in rational emotive behavior therapy (REBT), aimed at treating binge eating behavior among female college students. The results show that the program was effective, with positive effects on participants’ self-esteem, covert narcissism, perfectionism, body dissatisfaction, anxiety, depression, and binge eating.

## Background

Over the past two decades, South Korean society has increasingly pursued values based on physical appearance. Consequently, women with normal body weight, particularly college students, have distorted body images, are dissatisfied with their physical appearance, and idealize the thin body type, resulting in inappropriate eating habits and high likelihood of developing eating disorders [1]. According to one study, 37.75% of female college students in South Korea have been shown to be at risk of developing eating disorders, with 38.6% misguidedly considering themselves overweight, leading to problems with their dietary behavior [2]. Moreover, the incidence of bulimia nervosa, an eating disorder, has increased, and women are 15 times more likely than men to develop this disorder. It should also be noted that 44.9% of those treated for bulimia nervosa in South Korea are in their twenties [3].

According to the Health Insurance Review & Assessment (HIRA) Service, the incidence of eating disorders in Korea rose by 18.8% over 5 years from 2008 to 2012, with an annual increase of 4.5%. Of all those diagnosed with eating disorders, 49.2% are between their teens and 30s, highlighting the gravity of the problem among the younger generation. In another study, over 50% of female students attempted to manage their body weight through dieting; more female students are at risk for developing an eating disorder compared to their male counterparts [4]. One study observed that the percentage of female students exhibiting abnormal eating behaviors, such as restrained and binge eating, is consistently on the rise, from 11.3% in 2009 to 21.5% in 2014 [5].

Binge eating behavior is characterized by both the amount of food consumed and loss of control [6]. Binge eating behavior occurs as a central symptom of bulimia nervosa (BN), binge eating disorder (BED), and the binge-purge type of anorexia nervosa (AN-BP) [6]. Binge eating disorder is a mental disorder; binge eating behavior can be seen as a behavioral symptom of and precursor to binge eating disorder [7]. Subjects with an eating disorder often hide their problems and do not ask for expert help, and the disorder seriously impairs the individual’s physical and psychosocial functions owing to incorrect eating attitudes [8]. In addition, eating disorders have a high mortality rate among mental disorders, a low recovery rate, and a high risk of recurrence [9]. With such a rapid rise in binge eating behavior—a stage preceding an eating disorder—an intervention for the prevention of eating disorders needs to be developed, as people with eating disorders may develop other physical, mental, and social problems [10]. Particularly, interventions for female students are crucial, as they are at the highest risk for binge eating behaviors [11].

Binge eating behavior is particularly influenced by negative psychological factors [12]. Women who exhibit a binge eating pattern have a stronger desire for recognition, are more sensitive to rejection or criticism, and place greater value on others’ opinions relative to women who do not display such behavior [13]. Cognitive factors, including self-esteem, covert narcissism, perfectionism, and body dissatisfaction [14–17], and emotional factors, including anxiety and depression [18], could partially explain binge eating behavior.

In particular, low self-esteem related to body image and outer appearance is reported to be a more potent predictor of binge eating behavior in women than in men [19]. Gordon and Dombeck [20] categorized narcissism as overt and covert narcissism and showed binge eating behavior to be strongly associated with covert narcissism. Covert narcissists have problems in interpersonal relationships, are sensitive toward others’ evaluation, and particularly, when they have a negative perception about their body, they demonstrate negative eating attitudes in order to be approved by others [21]. Maples et al. [22] also reported that covert narcissists exhibit abnormal eating behaviors. Perfectionism is a multidimensional construct, and it is characterized by striving to avoid an error or defect and meet the highest standards and expectations [23]. Excessive concerns and worrying about error constitute may induce binge eating behavior, and are reported as major predictors of binge eating behavior over time [24].

Women who exhibit binge eating behavior compare themselves to other women and idealize thin bodies, which is associated with their irrational belief that their value is determined by their weight or appearance. If these women fail to achieve that idealized image, they become dissatisfied with their bodies, which ultimately results in low self-esteem [25]. Anxiety is viewed as a state provoked by a sense of isolation or hostility arising from malfunctioning interpersonal relationships. Particularly, anxiety is an emotion that encompasses emotional aspects such as restlessness and worry as well as physical aspects such as avoidance and dizziness, and it is reported to be closely associated with binge eating [26].

The relationship between depression and eating problems has been consistently discussed [27], and particularly, people who engage in binge eating are reported to undergo more severe stress in their daily living and feel a sense of depression in their lives, which in turn induces binge eating [28]. Another study observed that people who feel more depressed report more binge eating or other mental disorders than those who do not [29].

Ellis [30] developed rational emotive behavior therapy (REBT) to mediate the irrational beliefs that cause emotional and behavioral problems. The cognitive-behavioral perspective holds that individuals who place excessive value on body shape and weight could develop binge eating behavior [31]. In this case, a simple REBT-based intervention with problem-oriented and directional characteristics could be used in the short term because it changes irrational beliefs into rational beliefs, ultimately altering negative emotions/behavior.

REBT has been used in various areas such as clinical psychology, education, and counseling [32], and its effectiveness has been demonstrated in psychotherapy, education, and counseling mediation, regardless of age, manner of delivery, or clinical symptoms [33]. Initially, REBT-based empirical studies largely ignored specific mental disorders and explored the therapy and its effects in pan-diagnostic treatments. Therefore, in the early stages of REBT development, there was a lack of research that objectively validated its use with specific diseases [32].

In this study, a binge eating behavior management program based on the REBT theory was developed for female college students, and its effects were tested on self-esteem, covert narcissism, perfectionism, body dissatisfaction, anxiety, depression, and binge eating behavior.

## Methods

### Ethical approval

The study was approved by the research ethics committee at Sun Moon University prior to data collection (SM-201608-025-2). Prior to their participation, the study’s aims and methods were explained to the participants, and they were informed about the voluntary nature of participation, and assured of confidentiality, after which written consent was obtained from each participant.

### Experimental design

This was a quasi-experimental study, following a nonequivalent control group pretest-posttest design. The sample size was based on an effect size of 0.5, which was derived from the results of a meta-analysis on the effects of a cognitive-behavioral counseling program [34]; a significance level (α) of .05; and power (1-β) of .80. As per G*Power 3.1.3, the experimental and control groups required a minimum of 17 participants each. To account for dropout, 48 students (24 in each group) were recruited. However, two students in the control withdrew during the posttest. Accordingly, the final sample included 24 and 22 participants in the experimental and control groups, respectively.

### Participants

Current students of S university in C city were recruited through an on-campus recruitment poster. Those who provided informed consent to participate in the study were enrolled. The participant group allocation was determined by flipping a coin (heads: experimental group; tails: control group). Participants were included if they met the following inclusion criteria: tendency to engage in binge eating behavior, as demonstrated by eating attitude scores between 88 and 120 on the Bulimia Test Revised (BULIT-R; see Binge eating section for a discussion of this instrument and its use in the current study); no physical or mental illnesses that could hinder effective communication; and no involvement in any regular binge eating programs or other related educational experiences. The exclusion criteria were as follows: display of improper reward behavior (e.g., self-induced vomiting, diuretics, abuse of prescription drugs or other medications, fasting, or excessive exercise); intellectual disability; substance abuse and/or dependence; diagnosis of any organic mental syndrome; and disabilities that affect education (e.g., hearing and vision impairments).

### Procedure

Data collection was conducted from November 1 to December 2, 2016 in the following order: pretest, experimental treatment, and posttest. The data collection methods and procedures are described in the following subsections.

#### Pretest

The study purpose was explained to all participants prior to participation. All participants provided written informed consent, after which they completed a structured questionnaire regarding general characteristics, and scales on self-esteem, covert narcissism, perfectionism, body dissatisfaction, anxiety, depression, and binge eating. The completed questionnaires were immediately collected.

#### Experimental treatment

The REBT program was administered to the experimental group. The experimental group attended eight sessions lasting 90–120 min twice per week, with Sessions 4–6 conducted in two groups. The researcher and a graduate student assistant administered the program with the treatment group. The researcher explained the program to the research assistant and trained him in three 1-h sessions regarding the required information, attitudes, and cautionary measures. The control group received education regarding binge eating in one session. During the intervention period, the experimental group was provided with a binge eating-related program, and after termination of the study, the control group was provided with the same intervention program to ensure fairness.

#### Posttest

Immediately after the eighth session, posttest questionnaires were distributed to both groups. The questionnaire included items regarding self-esteem, inner narcissism, perfectionism, body dissatisfaction, anxiety, depression, and binge eating. Both groups completed the questionnaires and received small gifts for their participation.

During the intervention period, the control group received a binge eating-related intervention program. Upon termination of the study, the control group received the intervention program developed in the current study to ensure fairness.

### Measures

#### Self-esteem

Self-esteem was assessed using Ahn’s [35] Korean adaptation of Rosenberg’s [36] Self-Esteem Scale. This instrument consists of 10 items, with 5 items for positive self-esteem and 5 items for negative self-esteem. Each item is rated on a 4-point scale from 1 “strongly disagree” to 4 “strongly agree.” The total score ranges from 10 to 40, and a higher score indicates higher self-esteem. The reliability of the study as measured with Cronbach’s α was .81 in the study by Yoo [37] and .86 in this study.

#### Covert narcissism

The Covert Narcissism Scale (CNS) developed by Kang and Jeong [38], which was based on Akhtar and Thomson’s [39] clinical characteristics of narcissistic personality disorder*,* was used to measure covert narcissism. Each item is rated on a 5-point scale from 1 “strongly disagree” to 5 “strongly agree.” The total score ranges from 45 to 225, and a higher score indicates greater covert narcissism. The reliability of the study as measured with Cronbach’s α was .93 in the study by Kang [40] and .89 in this study.

#### Perfectionism

Perfectionism was measured using a multi-dimensional perfectionism tool, which was first developed by Hewitt and Flett [41] and later adapted into Korean by Kim [42]. This instrument consists of 45 items, with 15 items for self-oriented perfectionism, 15 items for other-oriented perfectionism, and 15 items for socially prescribed perfectionism. Each item is rated on a 7-point scale from 1 “strongly disagree” to 7 “strongly agree.” The total score ranges from 45 to 215, and a higher score indicates greater perfectionist tendency.

The Cronbach’s α was 0.87 in a study conducted by Jeon [43] and 0.91 in the current study.

#### Body dissatisfaction

The Body Shape Questionnaire (BSQ), developed by Cooper and colleagues [44] and later adapted into Korean by Noh and Kim [45], was used to determine participants’ degree of interest in their body shape. The body dissatisfaction tool consists of 32 items about negative affectivity originating from a feeling of being obese and distortion of the current body weight. Each item is rated on a 6-point scale from 1 “strongly disagree” to 6 “strongly agree.” The total score ranges from 32 to 192, and a higher score indicates more frequent feeling of being fat and lower satisfaction with one’s body. The Cronbach’s α was 0.88 in the original study and 0.94 in the current study.

#### Anxiety

The State-Trait Anxiety Inventory developed by Spielberger, Gorsuch and Lushene [46], is a commonly used tool to assess anxiety. Kim’s Korean adaptation [47], standardized by Hahn, Lee, and Tak [48], was used in the current study. This tool consists of 20 items, and each item is rated on a 4-point scale from 1 “strongly disagree” to 4 “strongly agree.” The total score ranges from 20 to 80, and a higher score indicates greater anxiety. The Cronbach’s α was 0.92 in a study conducted by Park [49] and 0.93 in the current study.

#### Depression

Depression was measured using the Korean version of the Center for Epidemiologic Studies Depression Scale (CES-D) developed by Radloff [50] to facilitate epidemiological studies on depression in the general population. Chon and Rhee [51] standardized the CES-D for use in Korean studies. This tool consists of 20 items, and each item is rated on a 4-point scale from 1 “never” to 4 “every day.” The total score ranges from 20 to 80, and a higher score indicates increasing severity of depression. The Cronbach’s α was 0.91 in a study conducted by Cha [52] and 0.91 in the current study.

#### Binge eating

In their development of the Bulimia Test, Smith and Thelen [53] relied on DSM-III criteria to diagnose bulimia nervosa. Thelen and colleagues [54] later used the DSM-III-R diagnostic criteria to develop the BULIT-R. The current study used Yoon’s [55] version of the tool to measure binge eating. This instrument comprises 36 items assessing five domains: binge eating, emotion, vomiting, food, and body weight. Each item is rated on a five-point scale, and the total score ranges from 36 to 180, where a higher score indicates stronger bulimia nervosa symptoms. In Korean studies, a score of 88 or higher indicates binge eating tendencies, while a score of 121 or higher warrants diagnosis and treatment for eating disorder [55, 56]. The Cronbach’s α was 0.90 in a study conducted by Kim [57] and 0.67 in the current study.

### Treatment

Table 1 shows the components of the REBT program, which included the following steps: ensuring that participants understood binge eating (first session); identifying participants’ belief systems (second session); changing participants’ beliefs (third session); forming correct eating habits by achieving cognitive reconstruction (fourth session); focusing on emotional control through emotional confirmation and relaxation (fifth session); focusing on emotional expression (sixth session); attempting behavior modification through training on problem-solving skills (seventh session); and ensuring self-management (eighth session). In addition to the REBT program, the researcher conducted weekly private one-to-one interviews with group members by telephone and in person, to prevent a high dropout rate, which might have influenced treatment effects.

**Table 1:** Content of the rational emotive behavior therapy-based binge eating behavior management program

Component	Session	Topic	Content	Method
Cognitive Reconstruction	1st	Understanding binge eating	-Introduction to the program’s purpose and methods -Creating an alias -Understanding binge eating -Participants share their binging behavior -Discussion and evaluation -Assignment: Dietary diary	Lectures, presentation, discussion, practice, presenting the assignment
	2nd	Identifying belief systems	-Understanding types of beliefs -Distinguishing between rational and irrational beliefs -Self-talking practice -Assignment: Dietary diary -Discussion and evaluation	Lectures, discussion, presentation, practice, discussion
	3rd	Changing beliefs	-Understanding the ABCDE theory -Dispute practice -Applying the ABCDE theory -Discussion and evaluation -Assignment: Applying ABCDE theory to real life	Lectures, practice, role play, discussion, presenting the assignment
	4th	Forming correct eating habits	-Sharing diet experiences -Understanding the advantages and disadvantages of one’s diet -Identifying incorrect eating habits -Understanding and planning correct diet habits -Writing to your future self (10 years later) -Discussion and evaluation	Lectures, discussion, presentation, practice
Emotional Control	5th	Emotional confirmation and emotional relaxation	-Identification of positive and negative emotions -Understanding and applying rational emotional imagery -Muscle relaxation training -Discussion and evaluation -Assignment: Applying rational emotional imagery to real life	Lectures, discussion, practice, demonstration, training, presenting the assignment
	6th	Emotional expression	-Understanding and practicing “I-Message” for emotional expression -Discussion and evaluation -Assignment: Applying “I-Message” to real life	Lectures, discussion, practice, role playing, presenting the assignment
Behavior Modification	7th	Problem solving training	-Identifying own problems -Finding ways to troubleshoot -Accepting less than perfect results -Discussion and evaluation -Assignment: Creating a problem-solving paper	Lectures, discussion, practice, role play
	8th	Self-management	-Finding ways to cope with a binging situation -Program evaluation and wrapping up -Share a rolling paper	Discussion, presentation, compensation (presenting gifts)

A *discussion and evaluation* component was included at the end of each session, to allow participants to present their thoughts and feelings and develop confidence through mutual support and encouragement. This section of the program was designed to reflect participants’ opinions regarding each session and improve program quality. Participants were asked to record the times and frequencies of their mealtimes; their thoughts, feelings, and behavior before/after the meal; and whether they were binging, in a *dietary diary,* to measure their eating habits and binging frequency. The participants were asked to write down a list of activities they liked and activities they did not like. Then, they were instructed to reward themselves with activities they liked when they did not binge eat, and engage in activities they did not like after binge eating.

Participants decided to enforce either compensation (e.g., watching movies or shopping for clothes) or punishment (e.g., exercising for 1 h or running for 1 h) based on their own assessments of how well they maintained their dietary records. In the final session, participants presented their dietary diaries, and the researcher used a reinforcement technique involving awarding gifts to participants who had made regular notes and practiced compensation and punishment well.

### Statistical analysis

SPSS 20.0 software was used to analyze the data (IBM SPSS Statistics for Windows, Armonk, NY: IBM Corp.). The measurement tools’ internal consistency was calculated using the estimated Cronbach’s α. A frequency analysis was performed to analyze participants’ general characteristics, and χ^2^ tests and t tests were performed in crosstabs analysis to assess homogeneity between the control and experimental groups. The Shapiro-Wilk test was used to assess the normality of the general characteristics and dependent variables. The normality of all variables at the baseline for the experimental group and control group was tested with skewness and kurtosis. The absolute skewness was smaller than 3, while the absolute kurtosis was smaller than 10, thereby satisfying the assumption of normality.

A *t*-test was performed to determine whether the self-esteem, covert narcissism, perfectionism, body dissatisfaction, anxiety, depression, and binge eating variables were homogeneous between the control and experimental groups. An analysis of covariance (ANCOVA) was performed to control for the pre-score difference between groups for variables that differed between groups in the pre-homogeneity test. The ANCOVA was also performed to verify the effect of the REBT program.

## Results

### Homogeneity test for general characteristics

The homogeneity test indicated no significant difference between the groups (*p* = .05); therefore, the two groups were homogeneous with regard to demographic variables (Table 2).

**Table 2:** Homogeneity of participants’ general characteristics (*N* = 46)

Division	Classification	Experimental group (*n* = 24)	Control group (*n* = 22)	*t*	*P-*value
		n (%)	n (%)		
Age	19–21	22 (91.70)	16 (72.70)	3.168	.205
	22–23	2 (8.30)	6 (27.30)		
	M ± SD	20.52 ± 0.79	20.91 ± 1.16	1.334	.189
Year	1–2	8 (33.30)	9 (40.90)	0.283	.595
	3	16 (66.70)	13 (59.1)		
Major	Nutrition, medical field	10 (41.70)	6 (27.30)	1.049	.306
	Non-nutrition, non-medical field	14 (58.30)	16 (72.70)		
Residence Type	House	10 (41.70)	7 (31.80)	0.406	.780
	Dormitory	9 (37.50)	10 (45.50)		
	Boarding/other	5 (20.80)	5 (22.70)		
Religion	Yes	9 (37.50)	6 (27.30)	0.546	.460
	No	15 (62.50)	16 (72.70)		
Weight (kg)	< 60 kg	14 (58.30)	14 (63.60)	0.136	.713
	> 60 kg	10 (41.70)	8 (36.40)		
	M ± SD	59.05 ± 8.70	58.30 ± 6.66	−0.322	.749

We assumed that certain general characteristics would affect binge eating behavior as religion influences food culture, and the degree of stress depends on an individual’s major. We also assumed that type of residence would have an effect on binge eating behavior; for example, a person living alone may have difficulty in forming regular eating habits.

Most participants in the experimental group (*n* = 24) as well as the control group (*n* = 22) were aged 19–21 years, at 91.7 and 72.7%, respectively. Regarding school year, 33.3% in the experimental group and 40.9% in the control group were 1st- or 2nd-year students. The percentage of students majoring in food and medicine was 41.7% and that of students majoring in non-food and non-medicine subjects was 58.3% in the experimental group; the percentages were 27.3 and 72.7%, respectively, in the control group. Regarding living arrangements, 41.7% of participants lived in their homes, 37.5% in the dorm, and 20.8% in a boarding house or other in the experimental group; the percentages were 31.8, 45.5, and 22.7%, respectively, in the control group. Regarding religion, in the experimental group, 37.5% identified themselves as religious, while 62.5% did not; in the control group, 27.3% were religious, while 72.7% were not. In the experimental group, 58.3% of participants weighed under 60 kgs, and 41.7% weighed 60 kgs or more, with a mean body weight of 59.05 kg. In the control group, 63.6% weighed under 60 kgs, and 36.4% weighed 60 kgs or higher, with a mean weight of 58.30 kg.

### Homogeneity test for the pre-experimental dependent variables

Table 3 shows the results of the pre-experimental homogeneity test for the dependent variables. The groups were homogeneous in the case of self-esteem, covert narcissism, perfectionism, physical dissatisfaction, anxiety, and depression. The variable of binge eating, however, was not homogenous, as the means (according to a five-point scale) for this item differed significantly (experimental group M = 93.83, SD = 8.42; control group M = 99.45, SD = 9.94).

**Table 3:** Homogeneity test for pre-experiment dependent variables (*N* = 46)

Variable	Experimental Group (*n* = 24)	Control Group (*n* = 22)	*t*	*P-*value
	M ± SD	M ± SD		
Self-Esteem	26.79 ± 3.86	26.09 ± 4.85	−.545	.589
Covert Narcissism	133.33 ± 19.14	135.00 ± 20.39	0.286	.776
Perfectionism	178.04 ± 29.59	183.23 ± 26.47	0.624	.536
Body Dissatisfaction	4.16 ± 0.84	133.50 ± 26.75	0.058	.954
Anxiety	48.46 ± 10.56	50.64 ± 11.93	0.657	.515
Depression	41.54 ± 10.56	44.45 ± 10.11	0.974	.336
Binge Eating	93.83 ± 8.42	99.45 ± 9.94	2.075	.044*

### Verifying program effectiveness

#### Effects on self-esteem

As illustrated in Table 4, the pretest and posttest results for self-esteem indicated that the experimental group’s posttest scores (M = 29.33, SD = 4.37) were higher relative to their pretest scores (M = 26.79, SD = 3.86), while the control group’s scores decreased from the pretest (M = 26.09, SD = 4.85) to the posttest (M = 24.77, SD = 4.57).

**Table 4:** Differences in dependent variables between groups

Variable	Group	Pretest	Posttest	*F*^a^	*P-*value
		M ± SD	M ± SD		
Self-esteem	Exp.	26.79 ± 3.86	29.33 ± 4.37	9.144	.004**
	Cont.	26.09 ± 4.85	24.77 ± 4.57		
Covert narcissism	Exp.	133.33 ± 19.14	122.25 ± 23.96	4.424	.041*
	Cont.	135.00 ± 20.39	138.18 ± 22.23		
Perfectionism	Exp.	178.04 ± 29.59	173.25 ± 30.31	1.52	.225
	Cont.	183.23 ± 26.47	182.91 ± 25.43		
Body dissatisfaction	Exp.	133.04 ± 27.01	115.17 ± 26.89	12.441	.001***
	Cont.	133.50 ± 26.75	144.23 ± 17.99		
Anxiety	Exp.	48.46 ± 10.56	41.25 ± 9.54	10.544	.002**
	Cont.	50.64 ± 11.93	50.91 ± 10.41		
Depression	Exp.	41.54 ± 10.16	37.21 ± 8.90	13.245	.001**
	Cont.	44.45 ± 10.11	47.32 ± 9.01		
Binge eating	Exp.	93.83 ± 8.42	69.67 ± 14.73	20.538	< .001***
	Cont.	99.45 ± 9.94	93.36 ± 16.13		

* *p* < .05, ** *p* < .01, *** *p* < .001

^a^ Measured by an analysis of covariance, *Exp.* Experimental group, *Cont.* Control group

#### Effects on covert narcissism

Results for covert narcissism showed that the experimental group exhibited a decrease in scores from pretest (M = 133.33, SD = 19.14) to posttest (M = 122.25, SD = 23.96). However, the control group’s scores increased from pretest (M = 135.00, SD = 20.39) to posttest (M = 138.18, SD = 22.23; Table 4).

#### Effects on perfectionism

Regarding pretest and posttest perfectionism scores, the change in the experimental group’s scores (4.79) was greater relative to that in the control group’s scores (1.04), but this difference was nonsignificant. The experimental groups perfectionism scores decreased from pretest (M = 178.04, SD = 29.59) to posttest (M = 173.25, SD = 30.31). Further, the control group’s scores decreased from pretest (M = 183.23, SD = 26.47) to posttest (M = 182.91, SD = 25.43; Table 4).

#### Effects on body dissatisfaction

The body dissatisfaction results indicated that the experimental group’s scores decreased from pretest (M = 133.04, SD = 27.01) to posttest (M = 115.17, SD = 26.89). However, the control group’s scores increased from pretest (M = 133.50, SD = 26.75) to posttest (M = 144.23, SD = 17.99; Table 4).

#### Effects on anxiety

The experimental group’s anxiety scores decreased from pretest (M = 48.46, SD = 10.56) to posttest (M = 41.25, SD = 9.54), while the control group’s anxiety scores increased from the pretest (M = 50.64, SD = 11.93) to the posttest (M = 50.91, SD = 10.41; Table 4).

#### Effects on depression

The experimental group’s depression scores decreased from pretest (M = 41.54, SD = 10.16) to posttest (M = 37.21, SD = 8.90). However, the control group’s scores increased from the pretest (M = 44.45, SD = 10.11) to the posttest (M = 47.32, SD = 9.01; Table 4).

#### Effects on binge eating

The difference between the pretest and posttest scores in the experimental group (24.16) were greater relative to those in the control group (6.09). Scores decreased from pretest (M = 93.83, SD = 8.42) to posttest (M = 69.67, SD = 14.73) in the experimental group. In addition, the control group’s score on binge eating decreased from pretest (M = 99.45, SD = 9.94) to posttest (M = 93.36, SD = 16.13; Table 4).

## Discussion

The REBT-based binge eating behavior management program developed in this study reduced participants’ irrational beliefs, that is, the cognitive factors of covert narcissism, perfectionism, and body dissatisfaction, and the emotional factors of anxiety and depression, as well as the behavioral factor of binge eating, while positively impacting participants’ self-esteem. We discuss these results below.

First, after completing the REBT-based binge eating behavior management program, the experimental group had a significantly higher self-esteem score than the control group, thereby providing evidence for effectiveness of this program. Previous findings of Saelid and Nordahl [58], who applied a short three-session program using the ABC model of REBT, support our findings. However, our results are in contrast with the result of a previous study, where a cognitive behavioral program did not effectively increase self-esteem in female undergraduates [59], although that program’s effectiveness may have been impacted by its inclusion of a cognitive restructuring intervention in only one out of the eight sessions (specific methods were not included in the report of the earlier study). On the other hand, our program significantly increased self-esteem by converting irrational beliefs to rational ones by specifically using a technique known as refutation based on the ABCDE model. Further, checking the restructured cognition through role play and presenting a task in each session to help participants to practice it in their daily living seemed to have contributed to their increased self-esteem.

Second, after the REBT-based binge eating management program, the experimental group had a significantly lower covert narcissism score than the control group, thereby providing evidence for the effectiveness of this program. This is similar to previous results of reduction in social media addiction tendencies through cognitive therapy with a focus on maladaptive cognitive emotional control strategies [60]. Further, Jeon [61] also reported that mindfulness intervention, a type of cognitive behavioral therapy, for participants with high covert narcissism significantly lowered covert narcissism, which supports our findings. As people with high covert narcissism fear criticism and rejection and feel embarrassment, we believe that encouraging the expression of their emotions through the “expressing negative emotions” activity in session 6, and helping them forgive and accept themselves through the “accepting less perfect results” activity in session 7, would have facilitated the positive outcome.

Third, after the REBT-based binge eating management program, while the posttest mean perfectionism score was low in the experimental group, there were no statistically significant differences in perfectionism between the experimental group and control group. We assume that the participants’ experience of psychological pressure to see positive changes after the study could have contributed to lowering perfectionism, albeit nonsignificantly. In addition, considering that perfectionism is a continuous character trait that is difficult to be significantly altered even with therapeutic interventions [62], we can surmise that a significant difference could not be observed owing to the short intervention period. Subsequent studies should utilize more diverse intervention methods and periods to alter perfectionism and compare them.

Fourth, after the REBT-based binge eating behavior management program, the experimental group had a significantly lower body dissatisfaction score than the control group, thereby providing evidence for the effectiveness of this program. Previous studies on the effect of cognitive behavioral therapy on body satisfaction of female college students with a negative body image [63] and the effect of cognitive behavioral program and meditation on body image in female middle school students [64] support our results. The participants of our program exhibited abnormal eating behaviors due to their body dissatisfaction, such as going on a diet to attain a thin and beautiful body figure. The program taught them of the risk of binge eating and diet and attempted to convert their irrational belief favoring a slim body shape into a rational belief through refutations of notions such as “do you really have to be skinny?” “Is everyone skinny in the real world?” and “what are the benefits of being skinny?” Further, the “accepting less perfect results” activity attempted to lower participants’ dissatisfaction with their bodies. We believe these intervention techniques would have led to the positive outcome of lowering body dissatisfaction.

Fifth, after the REBT-based binge eating management program, the experimental group had a significantly lower anxiety score than the control group, thereby providing evidence for the effectiveness of this program. Previous results that REBT-based group counseling program was effective in lowering test anxiety in elementary school students [65] and high school students [66] support our findings. In this study, the addition of emotional control and behavioral correction interventions for cognitive restructuring rendered the program more effective in lowering anxiety. We also believe that directly intervening with emotional control would have contributed to significant improvement.

Sixth, after the REBT-based binge eating behavior management program, the experimental group had a significantly lower depression score than the control group, thereby providing evidence for the effectiveness of this program. As described in our review of previous studies, depression, in addition to anxiety, was an important predictor of binge eating. Our results are supported by a previous study in which cognitive behavioral therapy was found to be superior to supportive expressive therapy in lowering depression in patients with binge eating [67]. The study that observed that correcting irrational beliefs through a REBT-based group counseling program led to a significant reduction of depression Stice [68] also supports our findings. Fischer et al. [69] reported that the intervention group that underwent a cognitive behavioral group therapy showed significantly greater reduction in depression compared to the control group among binge eaters [70], and a study on the effects of cognitive behavioral therapy on depression in female college students with a negative body image Park and Son [63] also confirmed that the therapy is effective for female college students showing binge eating behaviors, which are in line with our study. Through this program, the participants examined negative thinking, and explored and honestly expressed their depressive emotions about themselves. Further, by using a rational emotive imagery technique as an intervention, the participants were capacitated to experience positive and healthy emotions instead of negative and inappropriate emotions that are provoked when thinking about a negative event. This intervention for emotional control seems to have contributed to the effectiveness of the program.

Seventh, the experimental group had a significantly reduced binge eating score than the control group in the posttest evaluation, thereby providing evidence for the effectiveness of this program. The participants noted in their evaluations that writing a diet journal to monitor their binge eating behaviors and their thoughts and emotions and rewarding and punishing themselves accordingly substantially helped them reduce binge eating behaviors. They also reported that the assignment that required them to continue practicing to refute their irrational beliefs after the end of the session was beneficial for altering their irrational beliefs, regulating negative emotions, and lowering binge eating. In this study, the emotional relaxation training in session 5 and expression of negative emotions and conversing with others without hurting their own or others’ feelings by using “I-message” in session 6 seem to have contributed to emotional control and ultimately to lowering binge eating.

Ultimately, these results provide evidence that the integrative cognitive, emotional, and behavioral techniques of REBT are appropriate and useful for intervention in binge eating behavior and bringing about positive changes in the factors involved. However, a few limitations need consideration. First, this study was conducted with female college students attending one university; therefore, the findings cannot be generalized. Second, as this program is an intervention for binge eating behavior and related factors, its ultimate goal is the prevention of eating disorders. In order to verify the effectiveness of the intervention in the long run, a long-term follow-up is needed, as the current study did not examine its long-term effects. Third, because the Cronbach’s α (=.67) for binge eating behavioral measures was low, other measures should be considered in future studies. Fourth, although we considered participants’ weight, more restrictive selection criteria (e.g., body mass index) should be considered in future. Finally, because the study was conducted using the nonequivalent control group design, the results have limited generalizability. Despite these limitations, this study is significant in that it is the first to apply REBT in a binge eating behavior intervention for young female students. Furthermore, by examining the risk factors of binge eating, it informs the direction subsequent studies may take to develop binge eating behavior intervention programs.

## Conclusions

In summary, the REBT-based binge eating management program for female college students positively altered their self-esteem, and led to reduction in covert narcissism, body dissatisfaction, anxiety, depression, and binge eating. This program was applied to female students, but we suggest that subsequent studies expand the target population according to age, sex, and degree of symptoms based on the intervention components of the program. Since the ultimate aim of this program is to prevent eating disorders, we suggest future researchers to conduct long-term follow-ups to examine whether the effects of the program persist after its completion. In addition, while a group counseling technique was used in this study, subsequent studies may develop and assess interventions using social media or mobile phone applications to enable ease of participation for those who cannot participate in person.

## Data Availability

The data sets analyzed in the current study are not publicly available, but is available from the corresponding author on reasonable request.

## Abbreviations

BULIT-R: Bulimia Test Revised
BSQ: Body Shape Questionnaire
CES-D: Center for Epidemiologic Studies Depression Scale
CNS: Covert Narcissism Scale
REBT: Rational emotive behavior therapy
